# Innate and Autoimmunity in the Pathogenesis of Inherited Retinal Dystrophy

**DOI:** 10.3390/cells9030630

**Published:** 2020-03-05

**Authors:** T. J. Hollingsworth, Alecia K. Gross

**Affiliations:** 1 Hamilton Eye Institute, Department of Ophthalmology, University of Tennessee Health Science Center, Memphis, TN 38163, USA; thollin1@uthsc.edu; 2 Department of Optometry and Vision Science, University of Alabama at Birmingham, Birmingham, AL 35294, USA; 3 Department of Neurobiology, University of Alabama at Birmingham, Birmingham, AL 35294, USA

**Keywords:** retinal degeneration, immunity, autoimmunity, rhodopsin, citrullination, retinitis pigmentosa

## Abstract

Inherited retinal dystrophies (RDs) are heterogenous in many aspects including genes involved, age of onset, rate of progression, and treatments. While RDs are caused by a plethora of different mutations, all result in the same outcome of blindness. While treatments, both gene therapy-based and drug-based, have been developed to slow or halt disease progression and prevent further blindness, only a small handful of the forms of RDs have treatments available, which are primarily for recessively inherited forms. Using immunohistochemical methods coupled with electroretinography, optical coherence tomography, and fluorescein angiography, we show that in rhodopsin mutant mice, the involvement of both the innate and the autoimmune systems could be a strong contributing factor in disease progression and pathogenesis. Herein, we show that monocytic phagocytosis and inflammatory cytokine release along with protein citrullination, a major player in forms of autoimmunity, work to enhance the progression of RD associated with a rhodopsin mutation.

## 1. Introduction

Inherited retinal dystrophies (RDs) are the result of mutations in genes associated with cells of the outer retina, primarily rod and cone photoreceptors and retinal pigment epithelium (RPE) [1]. RDs are often highly heterogenous in every aspect of the disease, from the age of onset to the rate of progression, to the very mechanisms underlying the pathogenesis [2]. In order to study the molecular mechanisms of pathogenesis underlying retinal disease, numerous animal models, mostly in mouse, have been generated, carrying genetic defects in several genes causing the disease. A major cause of RDs, specifically, autosomal dominant retinitis pigmentosa (RP), is the presence of dominantly inherited mutations in the gene for the rod photoreceptor protein rhodopsin [2]. While most rhodopsin mutations cause protein misfolding and subsequent apoptosis, the more severe mutations result in improper rhodopsin trafficking and can lead to a subsequent loss of outer segment formation [2]. These mutations result in improper localization of both normal and mutant rhodopsin to the cellular membrane of the inner segment, nuclear, and axonal regions of the rod photoreceptor cells [1]. Two rhodopsin mutations resulting in the earliest onset of retinal degeneration, rhodopsin Ter349Glu and Gln344Ter, behave in this manner [2]. In recent years, many studies have shown the involvement of the immune system in the pathogenesis of many retinal diseases including glaucoma, age-related macular degeneration (AMD), RP, and others [3,4]. Also brought to light is the role of autoimmunity in RDs including AMD and RP [3,5,6,7,8]. While the eye is immune-privileged, the homeostatic disruptions caused by the progression of RDs can allow for a loss of this immune-privileged status, mainly due to the breakdown of the blood retinal barrier and choroidal neovascularization. In this study, we show the involvement of pro-inflammatory pathways in the progression of rhodopsin-mediated RD using the Ter349Glu rhodopsin knock-in mouse model of the disease, including monocytic phagocytosis and activation of the Janus Kinase/Signal Transducer and Activator of Transcription (JAK/STAT) pathway. We also show the presence of known autoimmunity proteins and protein modifications in the rhodopsin Ter349Glu mutant mouse.

## 2. Materials and Methods

### 2.1. Measuring Electrical Function in the Ter349Glu Rhodopsin Knock-in Mouse by Electroretinogram (ERG)

Wild-type (*+/+*, WT), Ter349Glu rhodopsin heterozygous and homozygous mice at one month of age were dark-adapted overnight (O/N). The following day, the mice were anesthetized using ketamine/xylazine (14.3 mg/mL ketamine/2.8 mg/mL xylazine in PBS, pH7.4), and ERGs were performed. Dark-adapted flashes (505 nm stimulus) of varying intensities were achieved using neutral density (ND) filters of nominal optical densities (OD) 4.8, 1.4, 3.6, 2.4, 1.8, 1.2, 0.6, and 0.0. A dark-adapted green camera flash performed with no attenuation. Data were analyzed using Labview and IgorPro software.

### 2.2. Monitoring the Ter349Glu Rhodopsin Knock-in Mouse Retina for Vascular and Laminar Abnormalities

WT and Ter349Glu homozygous mice at 4 weeks of age were anesthetized using ketamine/xylazine (14.3 mg/mL ketamine/2.8 mg/mL xylazine in PBS, pH7.4). The mice were subsequently examined by optical coherence tomography (OCT) (Bioptigen 840 nm Spectral Domain-OCT, Durham, NC, USA) to measure retinal thickness and assess for structural anomalies. After OCT, the mice were injected intraperitoneally with 100 μL of 4% fluorescein, and the retinas were imaged approximately 1 to 2 minutes post-fluorescein injection by fluorescein angiography (FA), using a 488 nm lamp co-equipped with a Micron III digital microscope (Phoenix Laboratories, Mukilteo, WA, USA) to assess the retinal vasculature for abnormalities including neovascularization, retinal hemorrhage, and vessel alterations.

### 2.3. Immunohistochemical Survey for Inflammatory Markers in the C-Terminal Mutant Rhodopsin Knock-in Mouse Retina

Whole eyes from WT, Ter349Glu rhodopsin heterozygous and homozygous mice 4, 8, and/or 12 weeks of age were fixed in 4% paraformaldehyde (PFA) in PBS, pH 7.4, overnight at 4 °C, cryoprotected in 30% sucrose in PBS, pH 7.4, frozen in optimal cutting temperature medium, and cryosectioned into 10 μm-thick sections. After washing away the medium, sections were prepped for immunolabeling using heat-mediated antigen retrieval in 10 mM sodium citrate, pH 6.0, with 0.05% Tween-20 for 1 h. After cooling, the slides were washed in PBS and subsequently blocked in 10% goat serum/5% BSA/0.5% Triton X-100 in PBS for 1 h at RT. Following blocking, labeling for markers of retinal inflammation including activated macrophages (F4/80), phosphorylated STAT3 (pSTAT3), and suppressor of cytokine signaling 3 (SOCS3) was performed using fluorescent immunohistochemistry. For both pSTAT3 (Cell Signaling Technology, Danvers, MA, USA) and SOCS3 (abcam, Cambridge, United Kingdom) labeling, a goat anti-rabbit IgG secondary antibody conjugated to horseradish peroxidase (HRP) was allowed to bind the primary antibodies, and Cy3-Tyramide Signal Amplification (TSA, Perkin Elmer, Waltham, MA, USA) was used to visualize the proteins. Briefly, TSA uses the peroxidase activity of HRP to generate reactive oxygen species from peroxide. These reactive oxygen species then oxidize and covalently bind the Cy3-conjugated tyramide molecule to the nearby proteins including the antigen/antibody complex, thus amplifying the signal. Macrophage labeling was performed using an antibody to F4/80-antigen (Bio-Rad, Hercules, CA, USA) followed by a goat anti-rat IgG secondary antibody conjugated to AlexaFluor488 (Invitrogen, Carlsbad, CA, USA). All sections were co-labeled for rhodopsin using B6-30N or K62-82 (provided by W. Clay Smith, University of Florida, Gainesville, FL, USA) and goat anti-mouse IgG_1_ or IgG_3_ conjugated to AlexaFluor488 (Invitrogen). Labeling for Müller cells was performed using an antibody against glial fibrillary acidic protein (GFAP, EMD Millipore, Burlington, MA, USA) followed by goat anti-mouse secondary IgG_1_ conjugated to AlexaFluor647 (Invitrogen). All images were captured as Z-stacks and expressed as maximum intensity projections.

### 2.4. Analysis of Retinal Citrullination in the Ter349Glu/Ter349Glu Rhodopsin Mouse Retina by Fluorescent Immunohistochemistry

Whole eyes from 10- to 12-week-old mice were fixed in 4% PFA in PBS, pH 7.4, overnight at 4 °C, cryoprotected in 30% sucrose in PBS, pH 7.4, frozen in optimal cutting temperature medium, and cryosectioned into 10 μm-thick sections. After washing away the medium, the sections were treated using heat-mediated antigen retrieval by boiling in 10 mM sodium citrate, pH 6.0, with 0.05% Tween-20 for 1 h. After cooling, the slides were washed in PBS and subsequently blocked in 10% goat serum/5% BSA/0.5% Triton X-100 in PBS for 1 h at RT. Following blocking, the sections were incubated with primary antibodies against peptidyl arginine deiminase 4 (PAD4, ProteinTech, Rosemont, IL, USA) and citrullinated peptides (Clone F95, EMD Millipore, Burlington, MA, USA) O/N at 4 °C. After washing in PBS, the slides were probed using goat anti-rabbit IgG conjugated to AlexaFluor488 (Invitrogen) and goat anti-mouse IgM conjugated to AlexaFluor555 (Invitrogen), and the nuclei were labeled with DAPI (Invitrogen). After washing in PBS, the slides were mounted using Prolong Diamond Anti-Fade Mountant (Invitrogen) and imaged using a Zeiss 710 Laser Scanning Confocal Microscope (Zeiss, Oberkochen, Germany). All images were captured as Z-stacks and expressed as maximum intensity projections.

## 3. Results

### 3.1. Loss of Functional ERG in Early-Onset RD

**Figure 1:**
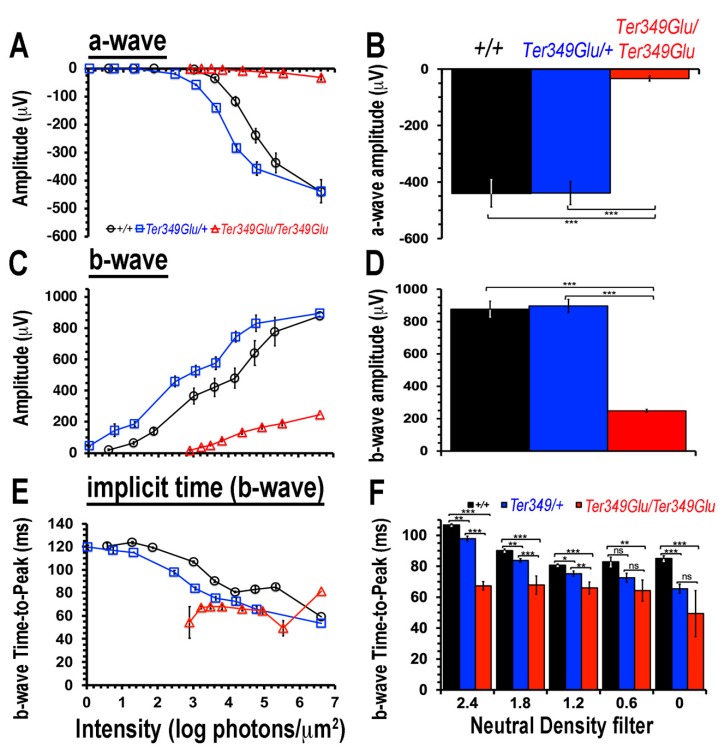
Electroretinogram (ERG) responses decline in mice expressing Ter349Glu rhodopsin. Using ERG to record extracellular potential differences across the retina, the electrophysiological function of +/+, *Ter349Glu/+*, and *Ter349Glu/Ter349Glu* mice was monitored by measuring a-, b-waves, and response latencies (Time-to-Peak, TTP) under increasing stimulus intensities (**A**,**C**,**E**). Graphs compare maximum average wave amplitudes and TTP (**B**,**D**,**F**) under dark-adapted conditions. Data analyzed using two-tailed T-test and expressed as the mean ± S.E.M. *, *p* < 0.05; **, *p* < 0.01; ***, *p* < 0.001; ns, not significant.

Patients expressing the Ter349Glu mutant rhodopsin experience a loss of photoreceptor function earlier in life, resulting in early and rapid central vision loss, when compared to other mutants of rhodopsin [9]. We tested if the Ter349Glu knock-in mouse displayed a similar early-onset (4 weeks of age) loss of visual capacity by ERG (Figure 1). Compared to +/+ mice (*n* = 5), the *Ter349Glu/Ter349Glu* mice (*n* = 5) showed an increase in the threshold of the dark-adapted b-wave by three orders of magnitude, with a maximum amplitude about 25% that of +/+ mice, while the maximum a-wave amplitude was only about 6% that of +/+ mice. This indicated a drastic loss of rod photoreceptor function. Interestingly, when compared to +/+ and *Ter349Glu/Ter349Glu* mice, *Ter349Glu/+* mice (*n* = 4) appeared to have a gain of function with an increase in sensitivity and a decrease in response latency without significant changes in amplitudes.

### 3.2. Effects of RD-Associated Photoreceptor Loss on Retinal Vasculature and Laminar Architecture

**Figure 2:**
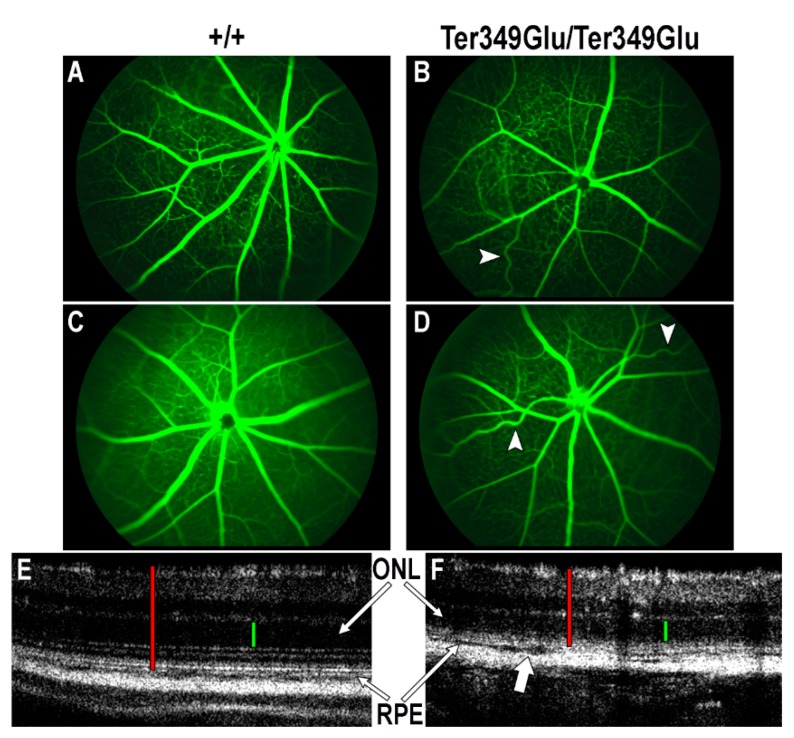
Ter349Glu rhodopsin knock-in mouse retina exhibits both vascular and laminar abnormalities. (**A**–**D**) Utilizing fluorescein angiography (FA), the state of the retinal vasculature of 4-week-old *+/+* (**A**,**B**) and *Ter349Glu/Ter349Glu* (**C**,**D**) mice was examined. Abnormal phenotypes varied in severity among mice, with overall attenuated retinal vessels and tortuous retinal vessels (arrowheads) being commonplace amongst all mice examined. (**E**,**F**) Optical coherence tomography (OCT) was used to examine the retinas of 4-week-old *+/+* (**E**) and *Ter349Glu/Ter349Glu* mice (**F**) for architectural abnormalities. *Ter349Glu/Ter349Glu* mice exhibited thinning of the outer nuclear layer (ONL) and patches of varying degrees of separation among the choroid, retinal pigment epithelium (RPE), and photoreceptors (block arrow), indicative of edema. Retinal thickness (red calipers) = 240 μm (**E**) and 180 μm (**F**); ONL (green calipers) = 60 μm (**E**) and 50 μm (**F**).

In cases of RP, retinal degeneration exerts effects on both retinal vasculature and laminar architecture in the forms of attenuated vessels and outer nuclear layer (ONL) thinning, respectively [10]. To examine the *Ter349Glu/Ter349Glu* retina for such abnormalities, FA and OCT were performed on 4-week-old +/+ and *Ter349Glu/Ter349Glu* animals in triplicate (Figure 2). Using FA, when compared to +/+ mice, *Ter349Glu/Ter349Glu* mice exhibited heterogeneous types and degrees of vascular abnormalities. The most common anomalies included attenuated vessels, tortuous vessels indicating hyperoxia, and reduced retinal venous and arterial vessel numbers. Using OCT, *Ter349Glu/Ter349Glu* mice exhibited the expected thinning of the ONL; however, at the interfaces between choroid, RPE, and rod outer segments (ROS)/ rod inner segments (RIS) layers the mice also exhibited a possible edema, likely due to loss of contacts between the RPE and the photoreceptors, associated with the lack of ROS. This edema was observed extending to varying degrees both inferiorly and superiorly to the optic nerve. Representative images were taken from the retina inferior to the optic nerve.

### 3.3. Activated Monocytes Are Present in RD Retinas from Rhodopsin Mutant Knock-in Mice

**Figure 3:**
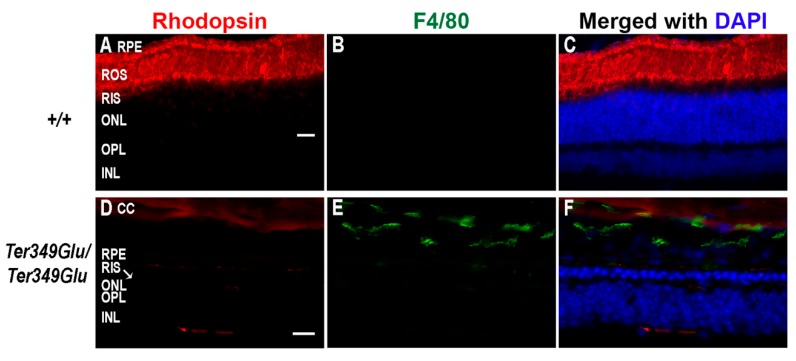
Retinas from retinal dystrophy (RD) mice exhibit monocyte activation. Using fluorescent immunohistochemistry, the presence of activated macrophages was examined in +/+ (**A**–**C**) and *Ter349Glu/Ter349Glu* (**D**–**F**) mice at 12 weeks of age. Retinal sections were labeled with anti-F4/80 antigen (green) and K62-82 (rhodopsin, red) antibodies. Nuclei were labeled with DAPI (blue). Autofluorescence in the choroid was observed in the red channel. CC, choriocapillaris; ROS, rod outer segments; RIS, rod inner segments; OPL, outer plexiform layer. Scale bars = 20 μm.

The retina contains resident macrophages similarly to the cortex, known as microglia, and these cells remain in the inner retinal layers under normal physiological conditions. Here, they remain in an inactivated state unless triggered by cytokine signaling or apoptotic signals [11]. Other types of leukocytes are typically not resident in the retina, and evidence of these cells in ocular tissues is indicative of retinal inflammation. Damage to retinal and choroidal vessels can allow leakage of not only blood-borne macrophages into the retina, but also cytokines, antibodies, and a plethora of other inflammatory factors [12]. Unfortunately, no method exists to differentiate between microglia and blood-borne macrophages; however, due to the observation of abnormal vasculature and retinal edemas, we chose to monitor the +/+ and *Ter349Glu/Ter349Glu* retinas for activated macrophages as a whole, using an antibody against F4/80 antigen, a cell surface glycoprotein expressed upon macrophage maturation (Figure 3). F4/80-positive macrophages were found in multiple animals from the *Ter349Glu/Ter349Glu* cohort at 12 weeks of age, with the most labeling observed in sections from *Ter349Glu/Ter349Glu* animals where nearly the whole retina was degenerated. Macrophages remained in the outer retina after almost total rod cell death (12 weeks). These macrophages were not observed in +/+ sections from any animal (*n* = 3 at all ages).

### 3.4. Activation of the Pro-Inflammatory JAK/STAT Pathway and Its Inhibitor SOCS3 in RD

**Figure 4:**
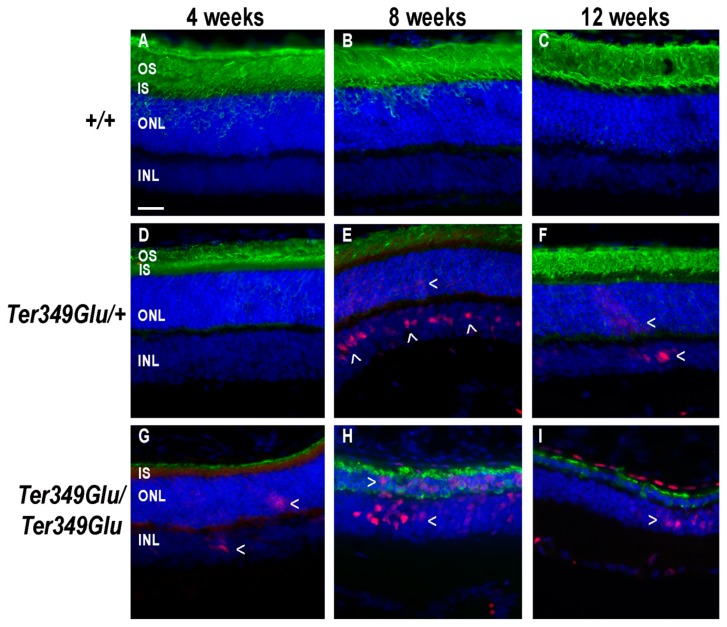
JAK/STAT pathway is activated in Ter349Glu rhodopsin knock-in mouse retina. Activation of the JAK/STAT pathway was examined using fluorescent immunohistochemistry on retinas from WT (*+/+,* **A**–**C**), Ter349Glu heterozygous (*Ter349Glu/+*, **D**–**F**), and Ter349Glu homozygous (*Ter349Glu/Ter349Glu*, **G**–**H**) mice. Retinal sections were treated for antigen retrieval and labeled for phosphorylated STAT3 (pSTAT3, red) and rhodopsin (green). Nuclei were labeled with DAPI (blue). Arrowheads (>), areas of JAK/STAT activation; OS, outer segments; IS, inner segments; INL, inner nuclear layer. Scale bar = 20 μm.

STAT3 is a downstream signaling partner of JAK. In inflammatory conditions, STAT3 is activated when cytokines such as IL-6, ciliary neurotrophic factor, leukemia inhibitory factor, and others bind to and activate the glycoprotein 130 (gp130) receptor [13]. Gp130 activates JAK, which in turn phosphorylates STAT proteins (pSTAT). Upon phosphorylation, pSTATs form both homo- and heterodimers, allowing nuclear entry and activation of gene transcription. The protein SOCS3 works as a negative regulator of the JAK/STAT pathway by binding the gp130 receptor and JAK together and blocking active sites involved in phosphorylation of STAT3, thus prohibiting further signal transduction [14]. In instances where inflammatory signaling needs to be slowed or stopped, SOCS3 works to perform this task. To examine the Ter349Glu knock-in mouse retina for inflammatory cytokine signaling, retinas from both +/+, *Ter349Glu/+,* and *Ter349Glu/Ter349Glu* mice were labeled with an antibody against pSTAT3 (Figure 4). We found that +/+ retinas showed no STAT3 activation across all ages, while the *Ter349Glu/+* and *Ter349Glu/Ter349Glu* mice exhibited increasing activation with age, beginning at 8 weeks and 4 weeks, respectively (*n* = 3 for all ages).

**Figure 5:**
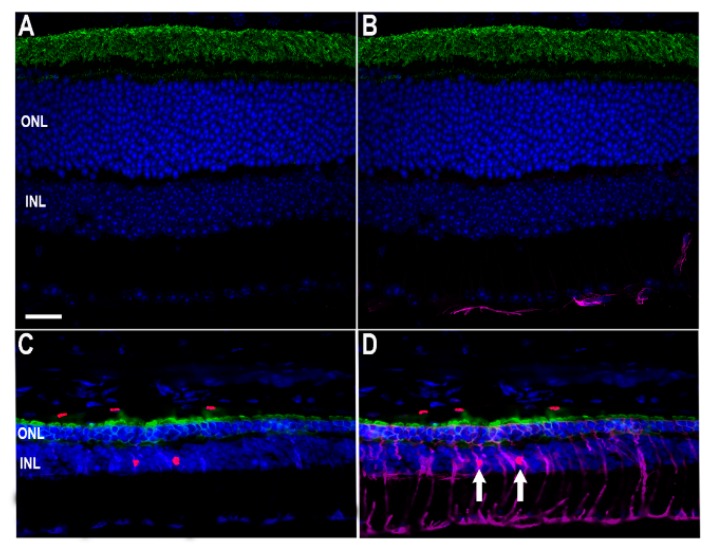
Identification of Müller cell nuclei as INL centers for STAT3 activation. To assess which retinal cells exhibited STAT3 phosphorylation, labeling was performed on +/+ (**A**,**B**) and *Ter349Glu/Ter349Glu* (**C**,**D**) animals for glial fibrillary acidic protein (purple), a marker of astrocytes and gliotic Müller cells, rhodopsin (green), and pSTAT3 (red). Nuclei were labeled with DAPI (blue). Arrows show red nuclei surrounded by purple. Scale bar = 20 μm.

Early pSTAT3 activation began in the inner nuclear layer (INL) and, by 12 weeks, extended to the RPE. The location of the activated nuclei in the INL suggested the nuclei belonged to Müller cells. Indeed, co-labeling for GFAP, a marker of gliosis in Müller cells, showed Müller cells to be pSTAT3-positive (Figure 5).

**Figure 6:**
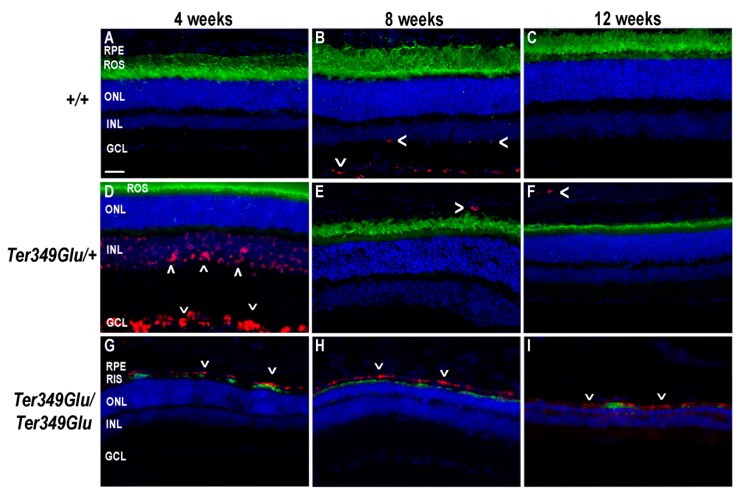
The JAK/STAT antagonist SOCS3 is expressed in Ter349Glu rhodopsin knock-in mouse retina. Using fluorescent immunohistochemistry, the expression of SOCS3, an antagonist to the JAK/STAT pathway, was examined in retinas from wild-type (WT, *+/+,* **A**–**C**), Ter349Glu heterozygous (*Ter349Glu/+,* **D**–**F**), and Ter349Glu homozygous (*Ter349Glu/Ter349Glu*, **G**–**I**) mice. Retinal sections were treated for antigen retrieval and labeled for SOCS3 (red) and rhodopsin (green). Nuclei were labeled with DAPI (blue). GCL, ganglion cell layer; arrowheads (<), SOCS3 labeling. Scale bar = 20 μm.

Since Müller cells maintain retinal homeostasis, this finding suggests they may act to respond to inflammatory cytokines, as STAT3 activation indicates the presence of pro-inflammatory cytokines in the neural retina, likely being released from activated macrophages within the retina. It should be noted that in these images, labeling for rhodopsin can be seen in the ONL even though rhodopsin is not normally localized in large amounts in this region. This is likely an artifact due to a heightened number of anti-rhodopsin epitopes following the antigen retrieval process. Labeling for SOCS3 revealed minimal expression in +/+ retinas while, similarly to pSTAT3, *Ter349Glu/+* and *Ter349Glu/Ter349Glu* mice began expressing SOCS3 in the neural retina and RPE at 4 weeks of age; with time, the expression increased in *Ter349Glu/Ter349Glu* mice and decreased in *Ter349Glu/+* mice (Figure 6, *n* = 3 at all ages).

### 3.5. Cell-Specific Expression of PAD4 and Heightened Citrullination in Early-Onset RD

**Figure 7:**
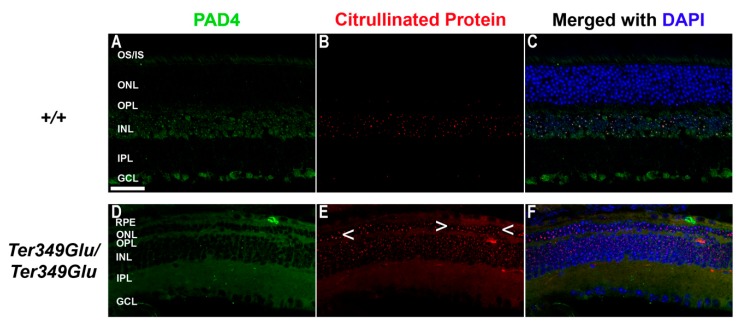
Expression of PAD4 and citrullination of retinal proteins in normal and degenerated states. WT (*+/+,* **A**–**C**) and *Ter349Glu/Ter349Glu* (**D**–**F**) mice at 10 to 12 weeks of age were labeled for PAD4 (green) and citrullinated peptides (red). Nuclei were labeled with DAPI (blue). Arrowheads (<), areas of increased citrullination in ONL; OS/IS, photoreceptor outer and inner segments; IPL, inner plexiform layer; Scale bar = 20 μm.

Recent studies have shown an increase in the expression of the deiminating enzyme PAD4 and increased citrullination in the event of ocular insult of the anterior segment [15]. Due to the inherent ability of citrullinated proteins to cause autoimmunity [16,17,18,19,20,21,22] and our previous finding that PAD4 is the primary retinal PAD in mouse [23], we tested the Ter349Glu retina for changes in PAD4 expression and citrullination compared with WT retina at 10 to 12 weeks of age (Figure 7). WT retina exhibited expression of PAD4 and exhibited INL nuclear citrullination, as shown previously [23]; however, the Ter349Glu retina exhibited higher levels of citrullination, much of it spanning the entire retina. This was observed in parallel with PAD4 expression increases, especially in the photoreceptors.

## 4. Discussion

Increasing amounts of evidence linking the immune system to ocular disease has emerged in the last decade. Studies focusing on glaucoma, AMD, RP, and other RDs have continued to show increased presence of many immune components including monocytes (blood-borne and resident), pro- and anti-inflammatory cytokines, and autoantibodies in many models of these diseases [24,25,26]. While all of these diseases present an initial insult of genetic and/or environmental origins, these findings overwhelmingly implicate the immune system in the pathogenesis of RDs. For example, the prevalence of single-nucleotide polymorphisms in the genes coding for complement factor H, complement factor I, as well as many other components of the innate immune system bolster the chances of developing AMD in otherwise normal patients [27,28]. In addition, models of glaucoma have shown the presence of macrophages and T-lymphocytes in the eye along with autoantibodies to proteins of the retina [3,4,29,30]. Models of RP have also shown similar features, including monocytic phagocytosis of both diseased and healthy retinal cells as well as numerous cytokines such as interleukin-1, interleuken-6, vascular endothelial growth factor, and others [4,31,32]. While the molecular mechanisms of the primary genetic assault have been teased out by copious amounts of work using cell culture and animal models for many RDs (i.e., loss of outer segment formation, disrupted visual cycle, etc.) [1,2,33], the full mechanisms of pathogenesis and progression of RDs still need thorough investigation before we fully understand them. In our findings, the *Ter349Glu/Ter349Glu* mice exhibited an almost complete loss of photoreceptor function by ERG; however, the *Ter349Glu/+* mice had heightened a and b waves while also showing a decrease in b wave latency compared to the *+/+* animals. This phenomenon might be explained by the expression levels of mutant rhodopsin compared to WT rhodopsin, as Ter349Glu rhodopsin expression levels are less than half of the WT levels [2]. This lower level of expression while still having 50% WT rhodopsin could result in a somewhat thinner outer segment with less rhodopsin protein in the discs, thus allowing for faster rates of diffusion of the phototransduction cascade components, decreasing the response latency, while normal WT rhodopsin activates the cascade, enhancing the signal. We also showed that animals with more advanced RD had significant numbers of macrophages, a feature found in many RDs, as well as activation of a known pro-inflammatory pathway (JAK/STAT). Interestingly, the *Ter349Glu/+* mice showed SOCS3 expression in the inner retinal layers early in life (4 weeks of age), with a dramatic decrease in expression with age, while the +/+ mice had minimal SOCS3 expression, which peaked at 8 weeks of age, and the *Ter349Glu/Ter349Glu* mice exhibited a somewhat constant SOCS3 expression in the outer retina and RPE. While the relatively stable SOCS3 expression in the *Ter349Glu/Ter349Glu* mice is explainable when compared to the levels of STAT3 phosphorylation which was present throughout the first 3 months of the animals’ life, the stark difference in SOCS 3 expression the *Ter349Glu/+* mice remains to be elucidated. More experiments examining the activation and deactivation of the proinflammatory JAK/STAT pathway are needed to better understand these expression differences. Further work will aim at pinning down the cytokines responsible for pathway activation; experiments inhibiting the pathway activation using antagonists to the JAK/STAT pathway and/or those inhibiting specific cytokine(s) will better underpin this pathway’s role in RD. Due to the nature of STAT3 role in numerous developmental pathways [13], a more targeted approach to delivering inhibitory compounds directly to the eye would be necessary. Future experiments will also work to decrease the number of activated macrophages in RD models to attempt to rescue some photoreceptor degeneration due to excessive phagocytosis and/or macrophage-derived cytokines. Work is already underway testing the inhibition of PAD4 in RD models to rescue retinal cells, retinal function, or both [34]. This work will be achieved using PAD4-deficient mice as well as inhibitors of the enzyme. Due to the excessive citrullination observed in *Ter349Glu/Ter349Glu* mice, it is not far-fetched to think that lowering or preventing this post-translation modification from occurring could lead to a slower rate of disease progression, allowing for not only longer lasting vision in patients affected but also an extended opportunity to correct the genetic insult, thus preventing further retinal degeneration. In all, our work further contributes to the increasing volume of studies indicating a role of the immune system in RDs as well as provides possible targets for the treatment of these debilitating blinding diseases.
